# Complete genome sequence of bacterium *Peribacillus simplex* strain IMGN11 from soil

**DOI:** 10.1128/mra.00643-24

**Published:** 2024-09-09

**Authors:** Sangmin Lee, Yeongjun Kim, Jeong A. Han, Ho-Seok Lee, Eun Yu Kim

**Affiliations:** 1 Department of Biology, Kyung Hee University, Seoul, South Korea; 2 Center for Genome Engineering, Institute for Basic Science, Yuseong-gu, Daejeon, South Korea; 3 Gyeonggido Agricultural Research & Extension Services, Hwaseong, South Korea; 4 Division of Natural and Applied Sciences, Duke Kunshan University, Kunshan, Jiangsu, China; 5 Environment Research Center, Duke Kunshan University, Kunshan, Jiangsu, China; The University of Arizona, Tucson, Arizona, USA

**Keywords:** *Peribacillus simplex*, plant growth promotion, bioremediation, biocontrol agent

## Abstract

We report the complete genome sequence of *Peribacillus simplex*, a spore-forming bacterium originally classified within the *Bacillus* genus. *Peribacillus simplex* exhibits antibiotic, plant growth-promoting, and xenobiotic-degrading activities and resistance to environmental contamination. The genome sequence of *Peribacillus simplex* will provide insights into its capabilities and potential as a biocontrol agent.

## ANNOUNCEMENT

*Peribacillus simplex* is a Gram-positive, nonpathogenic rhizosphere bacterium (1, 2). Due to its antibiotic activity (3), xenobiotic-degrading activity (4), plant growth promotion capabilities (5), and resistance to environmental contamination (2, 6), it has garnered attention for agricultural improvement. Moreover, its possession of endospores, which are resistant to heat, chemicals, and sunlight (7, 8), has prompted comparisons of its heat resistance and low-temperature growth with those of other bacteria. The primary objective of this genome announcement is to improve our understanding of its agricultural potential and suitability as a biocontrol agent.

The organism was isolated from the rhizosphere soil collected from the roots of cucumber plants at a depth of 10–15 cm at a cucumber farm located at (37°28'11.6"N, 127°37'59.3"E) in the Republic of Korea in 2019. After isolation, the soil was serially diluted from 1/100 to 1/1000 in distilled water (DW), and 100 µL was spread onto a tryptic soy agar (TSA) plate. Incubation was performed at 15°C for 16 hours in dark conditions, and the culture was streaked twice on new TSA plates to obtain a pure colony.

Genomic DNA was extracted from a single colony picked from the TSA plate using MagAttract HMW DNA Kit (QIAGEN, Cat No.67563) and quantified using a Qubit 2.0 fluorometer. To generate libraries, 5 µg of genomic DNA was sheared using the Megaruptor 3 according to the manufacturer’s protocols, and small fragments were subsequently removed by AMPure XP bead purification. The SMRTbell library was constructed using the SMRTbell Express Template Preparation Kit 2.0 (PacBio, Cat No. 100–938-900) according to the manufacturer’s instruction and sequenced using the Sequel I (Pacific Biosciences) sequencing platform.

The PacBio sequencing results for *Peribacillus simplex* IMGN11 produced 767,706,873 nucleotides and 659,201 reads with a mean coding sequence (CDS) length of 844.9 base pairs and an N50 value for the reads of 8,866 base pairs, resulting in a coverage of 582.63X. The assembly of reads was performed using SMRT Link 10.1.0 with the Microbial Assembly protocol, yielding two contigs: one is a complete genome comprising 5,706,799 base pairs and the other is a plasmid sequence of 5,963 base pairs (9). To improve the quality of the genome, we utilized automatic systems of read quality control, error correction, and adapter trimming within the PacBio SMRTLink system. The annotation with the NCBI Prokaryotic Genome Annotation Pipeline (PGAP) v6.7. predicted 5,341 protein-coding genes, 83 tRNA genes, and 39 rRNA genes. For more functional annotation, the predicted coding proteins were compared against protein family models (10, 11). Default parameters were used for all software, unless otherwise specified.

The complete genome of *Peribacillus simplex* strain IMGN11 comprises two contigs, one with a GC content of 40.1% and the other, representing a plasmid, with a GC content of 31.9%. These contigs include genes involved in the transport and metabolism of amino acids and inorganic ions. Furthermore, the genome harbors a high abundance of genes associated with energy production and conversion. Additionally, the CDSs were compared with the SWISS-Prot (12), KEGG (13), and SEED (14) databases using the UBLAST program (15). The potential of this bacterium as a biocontrol agent has been uncovered, given its abundance of genes associated with antibiotic activity, xenobiotic degradation, and plant growth promotion. Notably, 82 antibiotic-associated genes, including those related to antibiotic biosynthesis and resistance, were identified. Moreover, four xenobiotic-related genes, six cadmium-related genes, and three herbicide resistance genes were annotated. Additionally, nine genes responsible for the biosynthesis of plant growth-promoting substances, such as auxin and cytokinin, were identified. The availability of this genome sequence holds promise for uncovering novel factors contributing to biocontrol, xenobiotic activity, and bioremediation (16–19).

## Data Availability

The annotated genome sequence has been deposited in the NCBI under the GenBank accession number CP157082-CP157083. Additionally, the BioProject database accession number is PRJNA1100401, and the BioSample database accession number is SAMN40968167. The Sequence Read Archive information is available under the accession number SRR28779488.
